# CKM-YOLO11: A Lightweight Maize Foliar Disease Detection Model for Complex Natural Field Environments

**DOI:** 10.3390/s26102969

**Published:** 2026-05-08

**Authors:** Hui Zhu, Fulin Xiao, Jinfeng Xiang, Junting Guo, Hongbo Mu

**Affiliations:** College of Science, Northeast Forestry University, Harbin 150040, China; 2023213237@nefu.edu.cn (H.Z.); fulinxiao08@nefu.edu.cn (F.X.); xiangjinfeng@nefu.edu.cn (J.X.); 2024213132@nefu.edu.cn (J.G.)

**Keywords:** object detection, deep learning, maize foliar diseases, YOLO11, attention mechanism, lightweight model

## Abstract

Accurate and real-time detection of maize foliar diseases is important for field disease monitoring and yield protection. However, in complex natural field environments, different diseases often exhibit high visual similarity, and early weak lesions are easily confused with background elements such as dry leaves, soil, and shadows, leading to false positives and missed detections in existing models. To address these challenges, this study proposes an improved lightweight maize foliar disease detection model based on YOLO11, termed CKM-YOLO11. First, a mixed local channel attention mechanism is introduced and adapted to the task in the backbone to construct the C3k2-MLCA module, thereby enhancing joint modeling of local lesion textures, edge details, and global contextual information. Second, a lightweight residual attention module, named MLCA-HeadLite, is designed at the P5 layer of the neck/head to alleviate the suppression of weak lesion responses during deep feature fusion. Experimental results demonstrate that the proposed model achieves an mAP@50 of 81.5% on a self-constructed maize disease dataset with complex field backgrounds, improving mAP@50 and mAP@50–95 by 3.2 and 3.4 percentage points, respectively, compared with the baseline YOLO11, while maintaining a low parameter count and computational cost. Further analyses based on the confusion matrix, comparisons of detection results, and Grad-CAM visualizations indicate that the proposed model performs better in background suppression, retention of weak lesion responses, and robustness in complex scenes. This study provides a reference for the lightweight design of maize foliar disease detection models in complex field environments and their deployment on agricultural edge devices.

## 1. Introduction

Maize is a major food, feed, and industrial crop, and its stable production is of great importance for food security and the sustainability of agricultural production systems. In recent years, plant disease detection has been widely regarded as an important approach for improving agricultural productivity and reducing crop losses [1]. In maize production, foliar diseases have become one of the major factors affecting yield due to their wide distribution, high incidence, and direct impact on leaf photosynthesis [2,3]. Among these diseases, gray leaf spot is a major maize foliar disease worldwide and poses a serious threat to maize production [4]. Therefore, rapid and accurate identification of maize foliar diseases is of considerable practical value in agriculture [1].

Traditional maize disease identification mainly relies on manual field inspection and expert judgment, but this approach suffers from low efficiency, strong subjectivity, and limited consistency under large-scale cultivation conditions [2,3]. In terms of pathological morphology, northern leaf blight usually appears as fusiform or elongated elliptical lesions and exhibits distinctive pathological characteristics [5]. Gray leaf spot tends to expand along the leaf veins, forming elongated strip-like lesions [4]. Leaf rust typically manifests as densely distributed small pustules, with smaller lesion size and a more scattered distribution [2]. However, under actual field conditions, lesion areas are often visually confused with leaf senescence, local desiccation, vein textures, shadow occlusion, and soil backgrounds, making maize disease identification not only a classification task, but also a fine-grained detection problem in complex scenes [6].

With the development of computer vision and deep learning technologies, remarkable progress has been made in convolutional neural network-based plant disease identification. Systematic reviews have shown that deep learning has been widely applied to plant disease classification, detection, and segmentation, and has demonstrated strong potential for early disease identification [7]. Notably, public datasets such as PlantVillage are mainly collected under controlled conditions, whereas non-laboratory datasets such as PlantDoc better reflect the influence of complex backgrounds, occlusion, and illumination variation on model generalization [8,9,10]. This further indicates that field data are important for improving the real-world applicability of disease detection models. Compared with traditional pathogen diagnosis methods such as microscopic examination and PCR, image-driven approaches can directly extract disease features from leaf images and offer clear advantages in non-contact, rapid detection, and real-time deployment [1]. A dedicated review on maize diseases has also shown that convolutional neural networks are continuously driving maize disease detection toward higher accuracy and greater practical applicability [3]. However, previous studies have also noted that model performance still depends heavily on data quality, scene complexity, and generalization to real environments [7].

Most existing studies have been conducted on images collected under simple backgrounds or controlled conditions, whereas complex backgrounds, scale variation, occlusion, and illumination disturbances in real field environments remain important factors affecting model performance [10,11]. Studies on field-collected maize disease datasets further show that there are clear differences between field-acquired images and laboratory-condition images, and these differences directly affect the detection performance of models in practical scenarios [11]. Under complex background conditions, introducing attention mechanisms helps improve the model’s ability to focus on real lesion areas; however, existing methods still suffer from insufficient local detail modeling. Recent studies have attempted to integrate lightweight attention mechanisms into YOLO-based agricultural detection frameworks. For example, Shui et al. introduced MLCA into an improved YOLOv8 framework for flowering Chinese cabbage detection, while Fu and Zhang incorporated C2f-MLCA into a lightweight YOLOv8-based rice leaf disease detection model [12,13]. These studies demonstrate the potential of MLCA-related designs for enhancing local–global feature representation in agricultural vision tasks. However, their applications were mainly based on YOLOv8 and targeted different crop detection or disease detection scenarios. Therefore, how to further adapt MLCA to the YOLO11 architecture and maize foliar disease detection task, while simultaneously enhancing local lesion texture representation and suppressing background interference in complex natural field environments, remains worthy of further investigation [6].

Among numerous object detection frameworks, the YOLO series has been widely used in agricultural vision tasks because of its balance between detection accuracy and inference speed [14,15,16]. Recent studies on maize foliar disease detection have also shown that combining lightweight backbones with attention mechanisms within the YOLO framework helps achieve a more reasonable balance among detection accuracy, model size, and edge deployment efficiency [15,16]. In this study, YOLO11 released by Ultralytics was selected as the baseline model because of its mature engineering implementation, comprehensive task support, and good adaptability for edge deployment [17]. According to the official documentation, YOLO11 supports various computer vision tasks, including object detection, instance segmentation, classification, pose estimation, and oriented object detection [17]. Nevertheless, existing detection models still face two prominent challenges in complex natural field environments: first, there is substantial visual confusion between disease regions and background regions; second, the commonly used multiplicative attention mechanism in deep feature fusion may excessively suppress weak but real lesion responses [6].

To address these challenges, this study proposes CKM-YOLO11, a lightweight maize foliar disease detection model based on YOLO11. First, the Mixed Local Channel Attention (MLCA) mechanism is embedded into the C3k2 module in the backbone to construct C3k2-MLCA, enhancing joint modeling of local lesion textures, edge details, and global contextual information. Second, the lightweight residual attention module MLCA-HeadLite is designed at the P5 layer of the neck/head to alleviate weak lesion suppression in deep features. Finally, the proposed method is evaluated through deployment position experiments, joint ablation studies, comparisons with mainstream models, and visual analysis, while its application potential in disease severity grading and field management support is further explored.

## 2. Materials and Methods

### 2.1. Self-Collected Image Acquisition

**Figure 1:**
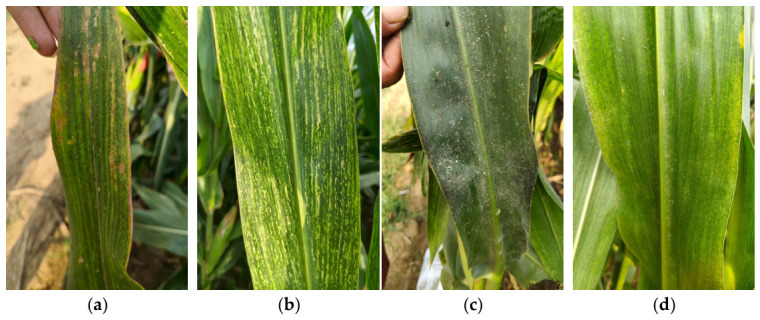
Representative smartphone-acquired maize leaf images in field conditions: (**a**) self-collected maize leaf image under natural illumination; (**b**) close-range field image showing leaf texture and background interference; (**c**) field-acquired maize leaf image with varied leaf posture; (**d**) maize leaf image collected under heterogeneous field-background conditions.

For the self-collected dataset, image acquisition was performed using a HUAWEI Mate 50 smartphone (Huawei Technologies Co., Ltd., Shenzhen, China) with a resolution of 2700 × 1224. Maize leaves were photographed in natural field environments under different illumination and background conditions. Close-range and multi-angle shooting captured lesion details. During collection, the shooting distance and angle were adjusted to reduce severe shadows, reflections, and blur, while moderate variations in illumination, leaf posture, background interference, and disease severity were retained to improve dataset diversity and realism. Representative field-acquired maize leaf images are shown in Figure 1.

### 2.2. Dataset Construction and Annotation

To improve model robustness in natural field environments, this study constructed a multi-source maize foliar disease detection dataset. The dataset was constructed using three public-data components together with a small number of self-collected maize leaf images. First, field-acquired maize disease images were obtained from the Corn Disease and Severity (CD&S) Dataset downloaded from OpenDataLab, and only the Dataset_Original folder in the raw dataset package was used (https://opendatalab.com/OpenDataLab/CD_and_S/tree/main, accessed on 3 May 2026). Second, to supplement the leaf rust category, additional images were collected from the train/Corn rust leaf folder of the PlantDoc-Dataset GitHub repository (https://github.com/pratikkayal/PlantDoc-Dataset/tree/master/train/Corn%20rust%20leaf, accessed on 3 May 2026). Third, leaf rust images from the test folder of the same PlantDoc-Dataset repository were also used (https://github.com/pratikkayal/PlantDoc-Dataset/tree/master/test, accessed on 3 May 2026). In addition, a small number of self-collected maize leaf images were included as negative samples and field-background supplements. Considering that the present study focuses on object detection under complex backgrounds rather than image-level classification under simple-background conditions, priority was given during sample screening to images exhibiting real field textures, natural illumination variation, and background interference. Previous studies have shown that controlled-environment datasets and non-laboratory datasets differ substantially in terms of background complexity, imaging conditions, and lesion appearance, and these differences directly affect model generalization in real-world scenarios [8,9,10,11]. Although the CD&S Dataset also contains augmented images, this study used only the original field-collected images from the Dataset_Original folder to avoid potential similarity-related data leakage [11].

**Figure 2:**
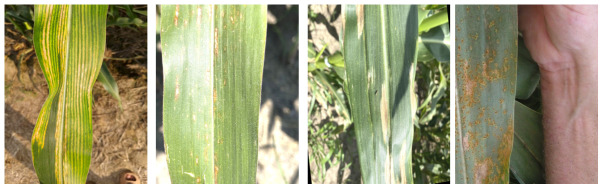

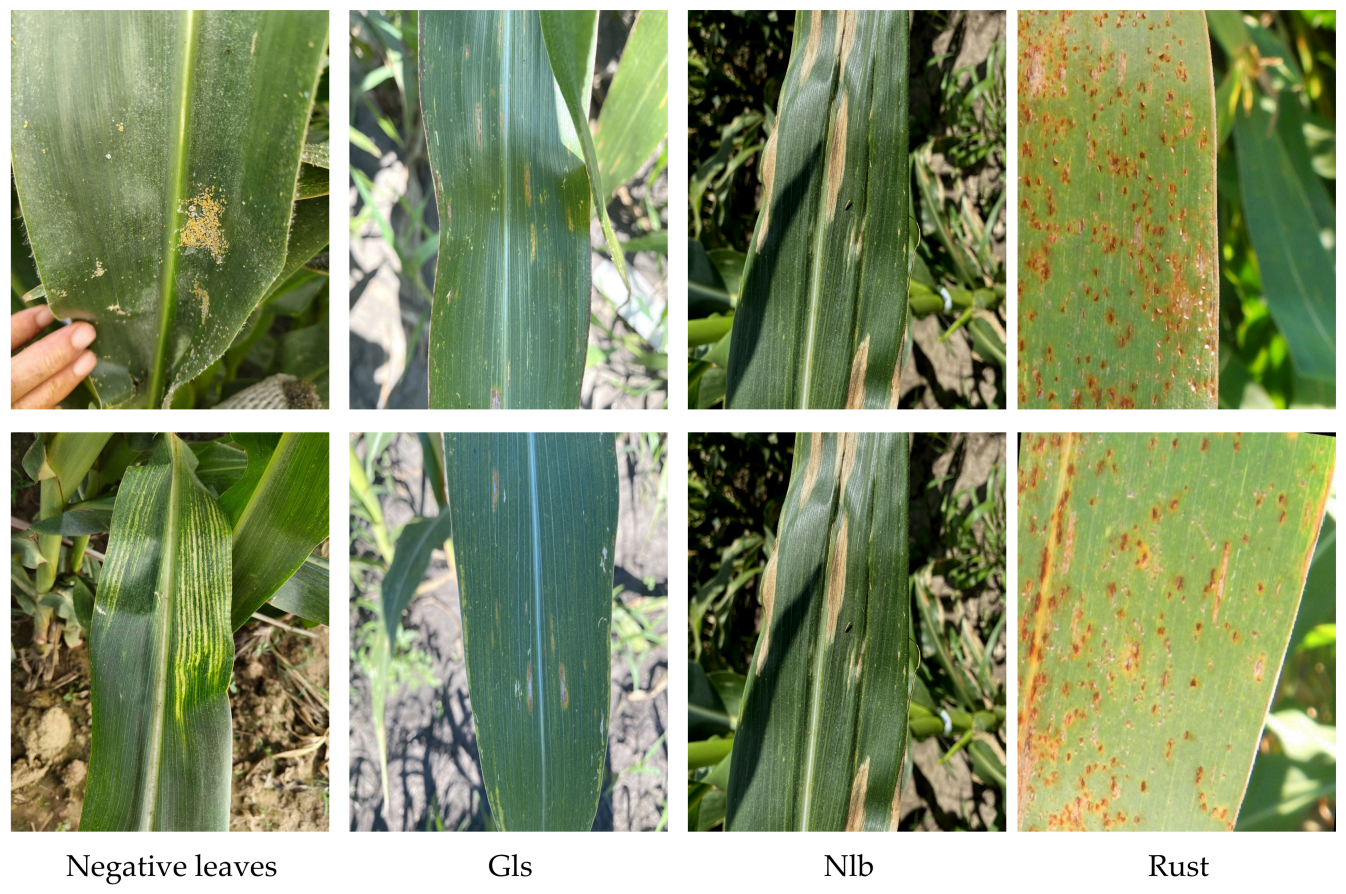
Examples from the maize foliar disease dataset: negative leaves, gray leaf spot (Gls), northern leaf blight (Nlb), and leaf rust (Rust).

With regard to specific data sources, the original dataset did not rely entirely on a single public dataset, but was composed of multiple sources. Specifically, the Corn Disease and Severity (CD&S) Dataset mainly provided images of gray leaf spot and northern leaf blight. Negative-leaf samples and part of the leaf rust images were obtained from maize leaf images photographed and collected by this study under natural field conditions. To further supplement the leaf rust category, additional publicly available images were obtained from the PlantDoc-Dataset GitHub repository and were used only for category supplementation. The acquisition procedure described in Section 2.1 applies only to the self-collected images, whereas public images were obtained from the corresponding dataset repositories and then screened and reorganized for this study. Representative examples of the four image categories are shown in Figure 2.

Accordingly, the original dataset comprised four image categories: negative leaves, gray leaf spot, northern leaf blight, and leaf rust. Specifically, the original dataset included 351 negative leaf images, 262 gray leaf spot images, 256 northern leaf blight images, and 107 leaf rust images.

These 976 images were regarded as the source-image dataset before subsequent slicing and augmentation. After source-image-level splitting and subset-wise processing, the final detection dataset size is reported in Section 2.3. All dataset images were uniformly annotated using the open-source annotation tool Labelme (version 5.8.3). Rectangular bounding boxes were used to label lesion regions, and each annotated region was assigned a corresponding disease category. The leaf images were categorized into four classes: negative leaves without lesion annotation, gray leaf spot (gls), northern leaf blight (nlb), and leaf rust (rust). Annotation files were first generated in JSON format and then converted into TXT files to meet the input format requirements of YOLO11.

### 2.3. Dataset Splitting and Data Augmentation

Before describing dataset splitting and augmentation, the overall data processing workflow should be clarified:

Dataset construction and annotation → Dataset splitting → Data augmentation.

This workflow strictly follows a “split-first-then-augment” principle to ensure that training, validation, and test sets remain fully independent, preventing any data leakage.

Original images were first divided into training, validation, and test sets at the source-image level. Subsequent processing, including slicing or augmentation, was performed independently within each subset. Images derived from the same original image were always kept in the same subset, and no original image or its derivatives appeared across different subsets. This strict same-origin isolation strategy minimizes the risk of data leakage caused by highly similar samples [18].

To mitigate class imbalance, targeted offline augmentation was applied mainly to the training set. Validation and test sets underwent only source-consistent high-resolution slicing when necessary, with all slices remaining within their original subset; no offline or online augmentation was applied to these subsets to ensure unbiased performance evaluation. Particularly, northern leaf blight and leaf rust categories, which had relatively few original samples, were expanded during the offline processing stage.

Offline augmentation operations were carefully selected to minimize impact on lesion texture, including random flipping, contrast adjustment, and high-resolution slicing, preserving the original morphological characteristics of the lesions. During model training, the YOLO11 online augmentation pipeline was employed only on the training data, incorporating Mosaic augmentation and HSV perturbation to simulate occlusion, overlap, illumination variation, and shadow interference in field environments. An early stopping strategy was applied to reduce overfitting: if validation performance showed no significant improvement over 50 consecutive epochs, training was automatically terminated. This pipeline improves model generalization to complex scenes while preserving lesion integrity [1,2,19].

After dataset construction and processing, the final detection dataset comprised 4070 images, including 1100 gray leaf spot images, 1569 northern leaf blight images, 1050 leaf rust images, and 351 negative leaf images.

### 2.4. Maize Foliar Disease Detection Model

#### 2.4.1. YOLO11

YOLO11 is an end-to-end single-stage object detection framework composed of three main components: the backbone, neck, and detection head. The backbone is responsible for hierarchical feature extraction from input images, the neck performs multi-scale feature fusion, and the detection head outputs object categories and bounding box locations. Compared with earlier YOLO-series models, YOLO11 further optimizes feature extraction and multi-scale fusion while maintaining high detection speed, thereby achieving a favorable balance among detection accuracy, model compactness, and inference efficiency. In the backbone, YOLO11 introduces convolutional aggregation modules such as C3k2 to enhance multi-scale feature representation. Meanwhile, the neck enables interactive fusion between deep semantic information and shallow detail features through multi-scale pathways, thereby improving detection performance for small objects and structurally complex targets. For the maize foliar disease detection task considered in this study, YOLO11 provides a solid foundation for real-time detection and can satisfy the dual requirements of speed and accuracy in natural field environments. Therefore, it was selected as the baseline model [17].

**Figure 3:**
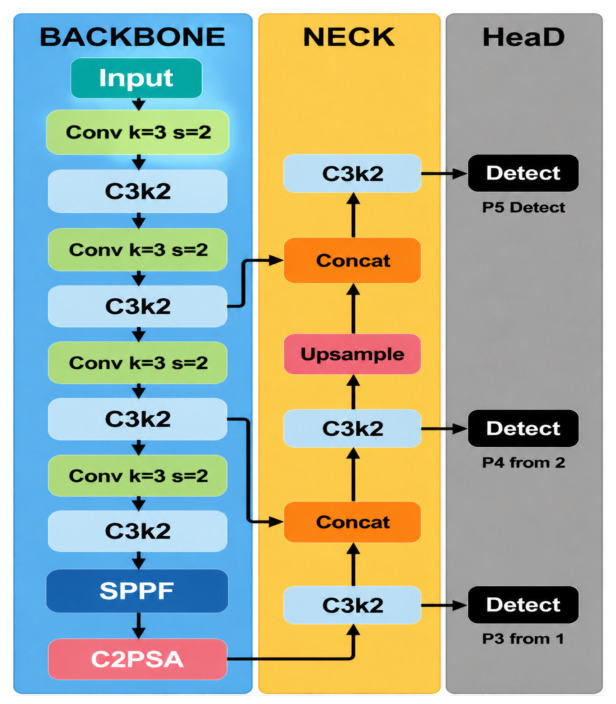
Baseline architecture of YOLO11. Arrows indicate feature-flow directions, and boxes denote the main functional modules in the backbone, neck, and detection head.

**Figure 4:**
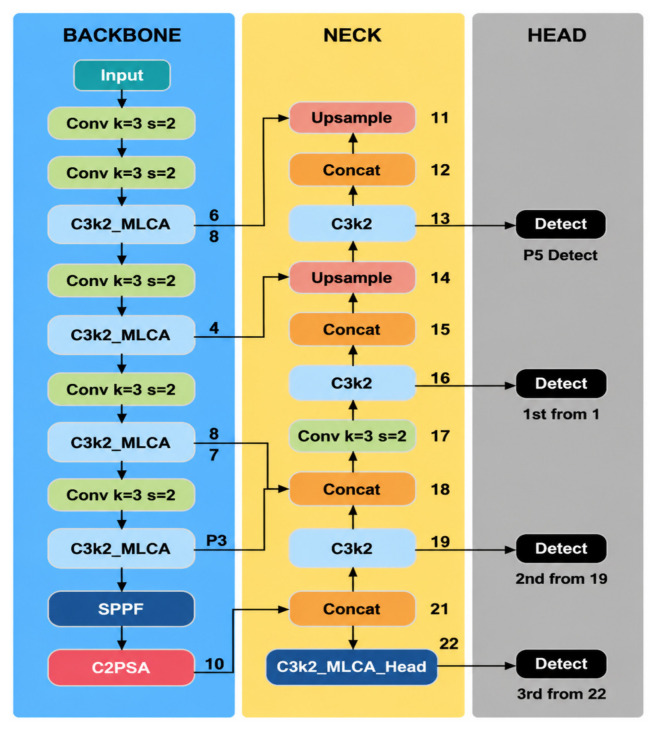
Overall architecture of CKM-YOLO11. Arrows indicate feature propagation, and boxes denote the improved C3k2-MLCA and MLCA-HeadLite modules and their positions in the network.

However, under complex field backgrounds, YOLO11 still faces two limitations. First, maize gray leaf spot, northern leaf blight, and leaf rust are often accompanied by interference factors such as dry leaves, shadows, vein textures, and soil backgrounds in natural environments, making it difficult for the model to stably capture subtle differences between lesions and background regions in shallow features. Second, during deep semantic feature fusion, traditional multiplicative attention mechanisms may excessively suppress weak lesion responses, thereby increasing the risk of missed detection. To address these limitations, targeted improvements were made based on YOLO11. First, MLCA was introduced into the C3k2 module in the backbone to construct the C3k2-MLCA module, thereby enhancing local–global joint modeling capability. Second, a lightweight residual module, termed MLCA-HeadLite, was designed at the P5 layer of the neck/head to alleviate the suppression of weak lesion responses in deep features. Based on these modifications, the improved model CKM-YOLO11 was developed. The baseline architecture of YOLO11 and the overall architecture of CKM-YOLO11 are shown in Figure 3 and Figure 4, respectively.

#### 2.4.2. Principle and Task-Oriented Implementation of Mixed Local Channel Attention (MLCA)

Mixed Local Channel Attention (MLCA) was originally proposed for object detection to combine local and global channel attention [20]. Existing channel attention mechanisms, such as SE, ECA, and CBAM, mostly rely on global average pooling (GAP) to extract channel descriptors [21,22,23]. Although these methods can enhance the responses of important channels, the strong compression of spatial dimensions tends to weaken local detail information during feature aggregation. This limitation is particularly evident in fine-grained lesion representation tasks under complex backgrounds, thereby restricting the model’s ability to characterize weak lesion edges, fine-grained texture differences, and small lesion regions in complex field environments [21,22,23,24]. To address this issue, this study introduces the Mixed Local Channel Attention (MLCA) mechanism and adapts it to the maize foliar disease detection task at the implementation level, so as to jointly model local spatial details and global channel semantic information while enhancing the network’s responsiveness to weak lesion regions.

**Figure 5:**
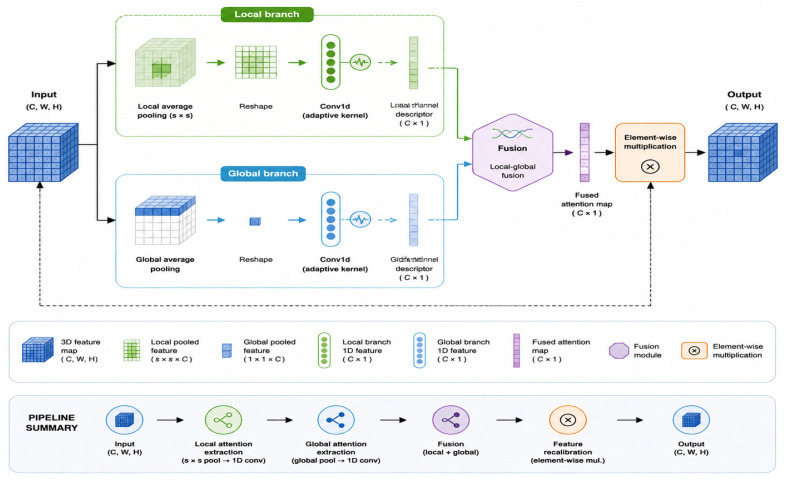
Schematic illustration of the Mixed Local Channel Attention (MLCA) mechanism, newly redrawn by the authors based on the methodological description of Ref. [20]. The local and global branches extract channel descriptors through pooling, reshaping, and one-dimensional convolution, and the fused attention map is used for feature recalibration.

**Figure 6:**
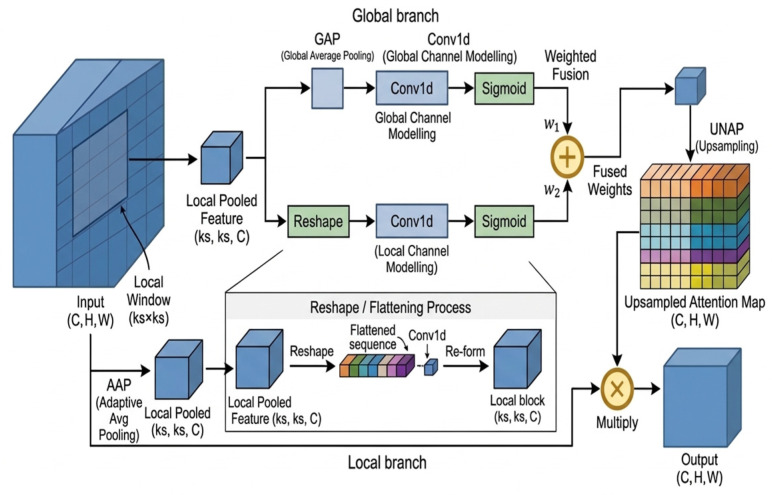
MLCA local–global joint modeling in this work. Boxes indicate the local and global attention branches, arrows indicate feature-flow directions, and the output attention map is used for feature recalibration.

A schematic illustration of the Mixed Local Channel Attention (MLCA) mechanism is shown in Figure 5. This figure was newly redrawn by the authors based on the methodological description of Ref. [20], rather than reproduced from the original publication. To better suit the maize foliar disease detection task, this study retains the core idea of local–global joint modeling while making implementation-level adjustments. Based on this task-oriented design, the MLCA module used in this work was constructed, as shown in Figure 6.

Specifically, given an input feature map $X\in R^{B\times C\times H\times W}$, where B denotes the batch size, C the number of channels, and H and W the spatial height and width, respectively, MLCA consists of two parallel branches: a global branch and a local branch. The global branch first applies global average pooling to the input features, compressing the spatial dimensions to 1 × 1 and generating a global channel descriptor. A one-dimensional convolution is then used to model cross-channel dependencies, followed by a Sigmoid activation function to produce global attention weights. In parallel, the local branch maps the input features to a fixed-size local-region representation through adaptive average pooling. The local region is then unfolded into a one-dimensional sequence, and a one-dimensional convolution is applied to model channel interactions within the local range. Finally, the output is reshaped back into a spatial form and passed through a Sigmoid activation function to generate local attention weights.

To balance representation capability and parameter overhead, the kernel size of the one-dimensional convolution is adaptively determined according to the number of input channels. Specifically, the intermediate variable t and the final convolution kernel size k are computed as follows:

(1) $$t=\left[\frac{\log_{2}(c)+b}{\gamma }\right]$$

(2) $$k=\left\{\begin{array}{cc} t, & t\;is\;odd\\ t+1, & t\;is\;even \end{array}\right.$$

where γ and b are hyperparameters controlling the channel-mapping ratio. Through this strategy, MLCA can dynamically adjust the channel modeling range in both the local and global branches according to different channel scales, thereby reducing parameter redundancy while maintaining good adaptability. In the implementation adopted in this study, the local weights generated by the local branch are denoted by $W_{1}$, and the global weight matrix is denoted by $W_{2}$. The two are linearly fused according to a preset weighting factor:

(3) $$W=\alpha W_{1}+(1-\alpha )W_{2},$$

where α denotes the local–global fusion coefficient. Rather than being manually preset as a fixed hyperparameter, α is a dynamically adaptive weight learned end-to-end by the network, and its contribution ratio can be automatically adjusted according to the spatial distribution and channel semantics of the input features. Unlike the original input feature size, the attention map generated by the local branch has a spatial size of s × s. Therefore, in the implementation of this study, the fused attention weights are mapped back to the original spatial size of the input feature map through adaptive pooling, and are then multiplied element-wise with the input features to complete feature recalibration, i.e.,

(4) Y=X·W

where · denotes element-wise multiplication, i.e., the Hadamard product rather than matrix multiplication. Through the above process, the module supplements fine-grained local responses while incorporating global channel contextual information, thereby enabling more sufficient recalibration of the input features. Compared with channel attention mechanisms that rely solely on global average pooling, this design helps enhance the model’s sensitivity to local lesion texture differences and weak-response regions, thereby improving the discrimination between diseased regions and interfering backgrounds under complex field conditions.

It should be noted that the MLCA used in this study is not a direct replication of the original mechanism, nor is it simply the same as previous YOLOv8-based MLCA applications. Previous studies have incorporated MLCA-related structures into improved YOLOv8 frameworks. Shui et al. introduced MLCA into the C2F module and combined it with a P2 detection layer, BiFPN, Wise-Inner-IoU, ByteTrack, and RGB-D-based depth estimation for multi-size, multi-target, and 3D position detection of flowering Chinese cabbage [12]. Fu and Zhang proposed a lightweight rice leaf disease detection model based on improved YOLOv8, in which ADown, C2f-MLCA, and DySample were introduced to improve disease feature perception and reduce computational burden under complex natural conditions [13]. These studies confirm the effectiveness of MLCA-related local–global feature modeling in agricultural vision tasks. However, the network architecture and task requirements of the present study are different. While preserving the core idea of local–global joint modeling, this study adapts MLCA to the C3k2 structure of YOLO11 and the characteristics of maize foliar disease detection. Specifically, MLCA is embedded into the C3k2 module of the YOLO11 backbone to strengthen the representation of lesion edges, fine-grained textures, and weak disease regions. In addition, a lightweight residual attention module, MLCA-HeadLite, is designed at the P5 layer of the neck/head to reduce the risk that weak lesion responses are excessively suppressed during deep feature fusion. Therefore, the proposed CKM-YOLO11 does not claim the first integration of MLCA into the YOLO family, but provides a YOLO11-oriented and maize-disease-oriented adaptation for complex natural field environments.

#### 2.4.3. C3k2-MLCA Module in the Backbone Network

The backbone is responsible for multi-scale feature extraction and serves as a critical stage for forming discriminative representations of lesion edges, local textures, and early weak features. Considering that maize foliar diseases are often visually confused with dry leaves, shadows, and vein textures in natural field environments, this study embeds MLCA into the C3k2 module of YOLO11 to construct the C3k2-MLCA module, thereby enhancing the backbone’s ability to jointly model local pathological features and global contextual information.

**Figure 7:**
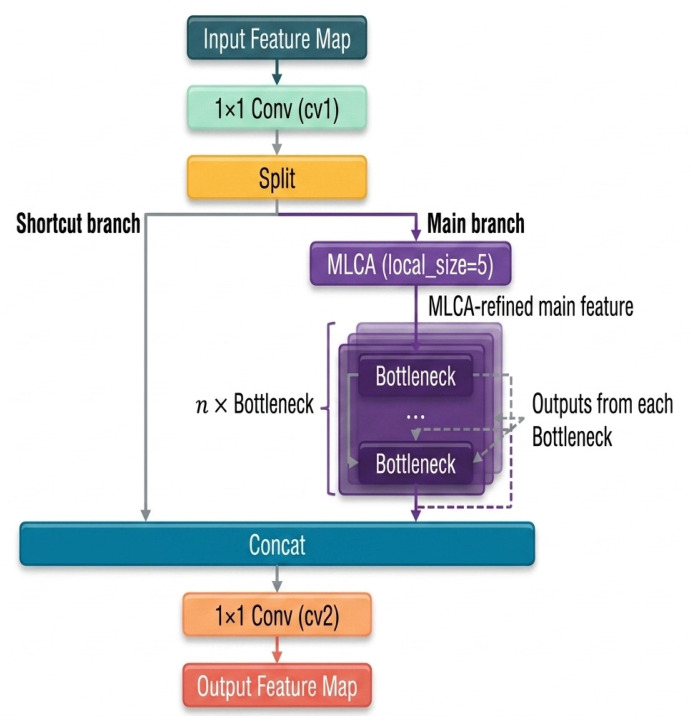
Schematic diagram of the C3k2-MLCA module. Arrows indicate data flow, and colored boxes denote the convolution, bottleneck, concatenation, and MLCA-based feature recalibration operations.

Structurally, the input features first pass through a 1 × 1 convolution for channel adjustment and are then divided into a main feature extraction branch and a cross-layer residual branch. To avoid excessive interference with the original residual pathway, MLCA is introduced only into the main feature extraction branch, while the cross-layer residual branch remains unchanged. Specifically, before being fed into the subsequent Bottleneck blocks, the main-branch features are first processed by MLCA for local–global information modeling and channel recalibration, and are then sent to the Bottleneck units for deeper semantic encoding. Finally, the outputs of the main branch and the residual branch are concatenated along the channel dimension, and a 1 × 1 convolution is applied to generate the module output. The core rationale behind this design is that C3k2 is located in the early-to-middle stages of backbone feature extraction, where the feature maps still preserve relatively rich spatial detail information. This makes the module more suitable for modeling lesion edges, texture variation, and local morphological differences. Embedding MLCA into the main branch, rather than at the output of the entire module, helps prioritize lesion-discriminative information in the main feature stream without disrupting the stability of the original residual pathway, thereby improving the separability between lesion and non-lesion regions under complex background conditions. The structure of the C3k2-MLCA module is shown in Figure 7.

In addition, considering that gray leaf spot and leaf rust often appear as elongated strip-like lesions or fine scattered spots in natural scenes, the local pooling size in this module was set to 5 to improve the network’s sensitivity to fine-grained lesion edges and local texture differences. Compared with channel attention mechanisms that rely solely on global pooling, this design is better suited to preserving the local morphological information of weak lesions during backbone feature extraction and provides more discriminative feature representations for the subsequent detection head.

#### 2.4.4. MLCA-HeadLite Residual Module in the Neck Network

**Figure 8:**
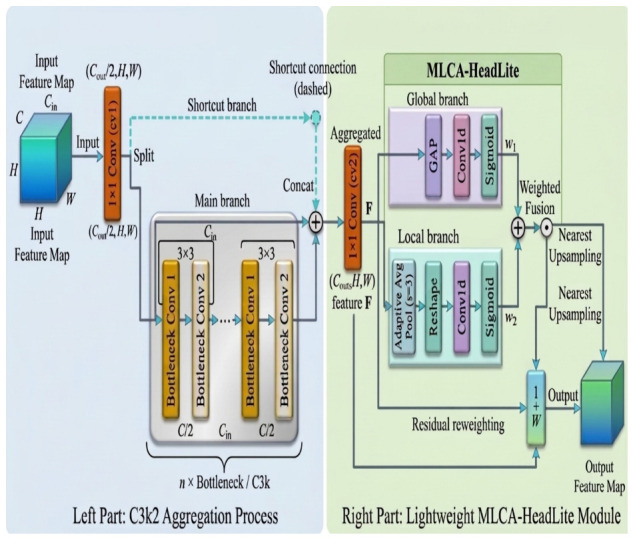
Schematic diagram of the MLCA-HeadLite residual module at the P5 layer. Boxes represent module operations, arrows indicate feature-flow directions, and the residual path indicates feature preservation for weak lesion responses.

At the P5 layer, where the deep neck and detection head intersect, the spatial resolution of the feature maps is substantially reduced and the semantic information becomes highly abstract. Conventional attention mechanisms generally adopt a standard multiplicative masking operation, i.e., Y=X·W. In such mechanisms, the input features are recalibrated element-wise by the attention weight map. While this operation can highlight salient regions, it may also amplify the suppression of genuine lesion features in weak-response scenarios [3,19,24]. In particular, when weak lesions exhibit high visual similarity to the background, the attention weights W generated by deep layers may approach zero. Under this condition, direct multiplication can completely suppress the already weak lesion responses, causing them to be submerged in background noise and thereby increasing the risk of missed detections. The MLCA-HeadLite residual module at the P5 layer is shown in Figure 8.

To address deep feature degradation at the P5 layer, this study designs a lightweight MLCA-HeadLite module equipped with a feature-preservation mechanism. First, to accommodate the relatively small spatial resolution at the P5 layer, the local pooling size in this module is reduced to S=3, and nearest-neighbor interpolation is used in place of more complex upsampling operations to reduce computational overhead. More importantly, instead of using the conventional multiplicative masking strategy, the proposed module adopts a residual reweighting formulation:

(5) $$Y=X\left(1+W\right).$$

In this formulation, the constant term 1 serves as a protective baseline for feature propagation. Even when the network assigns a very low attention score under complex background interference, i.e., W→0, the original input feature *X* can still be preserved and propagated to subsequent layers. This residual reweighting design allows the attention mechanism to function not merely as a multiplicative filter, but as a gentler feature enhancement strategy. As a result, even when the attention weights are low under complex background interference, the original input features can still be stably transmitted through the residual path, thereby reducing the risk that weak lesions will be excessively suppressed during deep feature propagation and improving the model’s robustness in complex field environments.

## 3. Experimental Results and Analysis

### 3.1. Experimental Settings and Evaluation Metrics

The hyperparameter settings used for model training are listed in Table 1, and the experimental environment is summarized in Table 2. All models were implemented, trained, and tested using Visual Studio Code (version 1.113.0; Microsoft Corporation, Redmond, WA, USA). Detailed training parameters and hardware/software configurations are provided below.

To comprehensively evaluate the effectiveness of the proposed lightweight model CKM-YOLO11 for maize foliar disease detection in complex field environments, Precision (P), Recall (R), Average Precision (AP), and mean Average Precision (mAP) were adopted as the primary evaluation metrics. To further assess the balance between precision and recall, the F1-score was also introduced and was automatically computed during the experiments. It is defined as the harmonic mean of precision and recall. Considering that this study targets deployment on agricultural edge devices, model complexity and runtime efficiency are also important considerations. Therefore, the number of parameters (Params) and floating-point operations (FLOPs) were used to evaluate the model’s spatial complexity and computational cost, respectively. In addition, frames per second (FPS) was adopted to measure the processing efficiency of the model in the complete detection pipeline. Specifically, FPS was calculated based on the total time consumed by preprocessing, inference, and postprocessing for a single image. The formulas for the above evaluation metrics are given as follows:

(6) $$Precision=\frac{TP}{TP+FP}\times 100\%$$

(7) $$Recall=\frac{TP}{TP+FN}\times 100\%$$

(8) $$AP=\int_{0}^{1}P(R)dR$$

(9) $$mAP=\frac{\sum_{i=1}^{C}AP_{i}}{C}\times 100\%$$

(10) $$F1=\frac{2PR}{P+R}$$

In the above formulas, True Positive (TP) denotes the number of maize disease samples correctly identified by the model; False Positive (FP) refers to background regions in complex field scenes, such as dry leaves or soil, that were incorrectly detected as lesions, or to incorrectly classified disease categories; and False Negative (FN) represents lesion samples missed because of weak early symptoms or severe background confusion. AP reflects the detection performance of a single category across different thresholds by jointly considering precision and recall, whereas mAP is the mean value of AP over all *C* categories and provides an overall measure of detection accuracy. The F1-score is used to characterize the balance between precision and recall.

**Figure 9:**
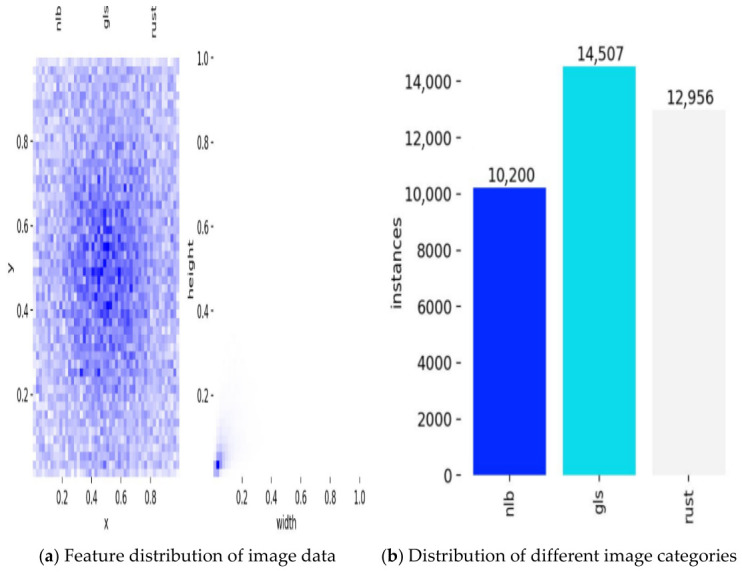
Statistical distribution of ground-truth bounding-box geometry in the dataset.

In addition to the above quantitative metrics, the geometric distribution of ground-truth bounding boxes in the dataset is also important for assessing the model’s generalization ability to lesions of different morphologies, as shown in Figure 9.

### 3.2. Comparison of MLCA-HeadLite Deployment at Different Detection Layers

To further investigate the adaptability of the lightweight attention module at different levels of the detection head, YOLO11 was used as the baseline model, and MLCA-HeadLite was deployed at P3, P4, P5, and several multi-layer combinations. Comparative experiments were then conducted in terms of detection accuracy, model complexity, and inference efficiency. The results are presented in Table 3.

From the single-layer deployment results, introducing MLCA-HeadLite into the detection head improved the overall performance of the model to varying degrees compared with the baseline. Among the single-layer settings, deployment at P3 improved Box, Recall, mAP@50, and F1-score to 0.798, 0.739, 0.808, and 0.770, respectively. This indicates that introducing a lightweight attention mechanism into shallow feature maps helps strengthen the representation of fine-grained textures, edge details, and small lesion targets. However, the inference speed dropped to 285.71 FPS, suggesting that although shallow-layer attention improves detection accuracy, it also incurs a certain loss in real-time performance. By contrast, the improvement achieved at P4 was relatively limited. Although the Box metric reached 0.799, no clear advantages were observed in Recall, mAP@50, or inference speed, while the parameter count and computational cost increased slightly. This suggests that deploying the module at the intermediate layer does not fully exploit the complementary effects of shallow detail information and deep semantic information, and its overall performance is inferior to that of the P3 and P5 settings.

Among the single-layer configurations, P5 achieved the best overall balance. Compared with baseline YOLO11, deploying MLCA-HeadLite at P5 improved Box, mAP@50, and mAP@50–95 to 0.804, 0.807, and 0.476, respectively, while the inference speed reached 344.83 FPS, which was not only higher than those of the P3 and P4 single-layer settings, but also exceeded that of the baseline model. This result indicates that introducing a lightweight attention mechanism at the P5 layer, where deeper semantic information is more concentrated, can enhance high-level semantic representation while maintaining strong detection efficiency. Therefore, P5 is a promising candidate for subsequent joint improvement.

From the results of multi-layer deployment, different combinations improved certain metrics, but the overall gains did not continue to increase with the number of deployment layers. For example, the P3 + P4 configuration achieved 0.780, 0.736, and 0.799 in Box, Recall, and mAP@50, respectively, which was overall inferior to deployment at P3 or P5 alone. The P3 + P5 configuration achieved a certain trade-off between Recall and inference speed, with mAP@50 = 0.801 and FPS = 333.33, indicating that jointly enhancing shallow detail features and deep semantic features has some potential. Although the P4 + P5 configuration achieved the highest Box value (0.809) among all comparison settings, its Recall and mAP@50–95 did not improve accordingly, and its overall performance remained less stable than that of P5 alone. These results suggest that deploying attention modules at multiple layers does not necessarily lead to better overall performance, and may instead weaken the gains because of feature redundancy and repeated enhancement.

Overall, the P3, P5, and P3 + P5 configurations all showed promising potential for improvement. Among them, P3 was more effective at enhancing shallow detail representation, P3 + P5 demonstrated a certain multi-scale collaborative advantage, and P5 achieved the best trade-off among detection accuracy, model complexity, and inference efficiency. Considering that the next stage further analyzes the synergy between head-side improvement and backbone-side improvement, the better-performing candidate settings were selected for the subsequent joint ablation study, with particular attention paid to the overall performance of P5.

These results further indicate that the lightweight attention module plays different roles on feature maps at different levels. The P3 layer has a higher spatial resolution and is therefore better suited to preserving tiny lesions and edge details, giving it advantages in Recall and mAP@50. In contrast, the P5 layer aggregates stronger high-level semantic information and is more suitable for recalibrating fused abstract disease features, thereby achieving a better balance among accuracy, complexity, and inference efficiency. This finding suggests that different feature levels play distinct roles in maize foliar disease detection, and that the deployment position of the attention module should be matched to lesion scale characteristics and scene complexity.

### 3.3. Ablation Study

In the previous experiments, the deployment effects of MLCA-HeadLite at different positions in the detection head were systematically compared. The results showed that the P3, P5, and P3 + P5 configurations all exhibited promising improvement potential, among which P5 achieved the best overall balance between detection accuracy and inference efficiency. However, the experiments in Section 3.2 only examined the effect of introducing the lightweight attention module on the head side alone, and therefore could not fully reveal its synergy with the feature enhancement strategy in the backbone.

To further evaluate the impact of different improved modules on the overall detection performance of YOLO11, and to analyze the effectiveness of joint optimization of the backbone and detection head, this study introduced the C3k2-MLCA module into the backbone based on the head-side screening results and constructed several improved model variants for ablation experiments. By comparing the performance differences among the baseline model, the model with only the head module, the model with only the backbone module, and the model with both modules, the specific contributions of each improvement strategy to detection accuracy, recall, inference speed, and overall efficiency can be more comprehensively revealed.

It can be seen from Table 4 that, after introducing different improvement strategies based on the baseline YOLO11, all detection metrics were improved to varying degrees. Among them, after adding MLCA-HeadLite at P5, the Box metric increased from 0.763 to 0.804, Recall increased from 0.719 to 0.725, and mAP@50 and mAP@50–95 increased to 0.807 and 0.476, respectively. With the parameter count remaining nearly unchanged, the detection speed further increased to 344.83 FPS, indicating that introducing a lightweight attention mechanism at the P5 position of the neck can effectively enhance high-level semantic representation while maintaining strong detection efficiency.

Furthermore, after embedding C3k2-MLCA into the backbone, the Box metric reached 0.808 and the F1-score increased to 0.770, indicating that strengthening local–global joint modeling during shallow feature extraction helps improve the model’s ability to capture lesion textures and edge information. After combining both improvements, the proposed CKM-YOLO11 achieved 0.747, 0.815, and 0.484 in Recall, mAP@50, and mAP@50–95, respectively, which represent improvements of 2.8, 3.2, and 3.4 percentage points over the baseline model. These results indicate that coordinated improvements in the backbone and neck can further enhance the overall detection performance of the model. Although the inference speed of the final model decreased to 294.12 FPS, it still maintained strong real-time performance, demonstrating that the proposed strategy achieves a reasonable balance between accuracy and efficiency.

The ablation study further indicates that the performance gains obtained in this study do not arise from the incidental accumulation of individual modules, but from the synergistic effect of coordinated optimization in both the backbone and detection head. Specifically, C3k2-MLCA mainly enhances joint modeling of local textures and global contextual information in the shallow stage, thereby improving the early discriminability of lesion regions, whereas MLCA-HeadLite alleviates the excessive suppression of weak lesion responses in deep features through a residual reweighting strategy. Together, these two modules improve maize foliar disease detection performance under complex background conditions.

### 3.4. Comparison with Other YOLO-Series Models

Table 5 summarizes the detection performance of different YOLO-series models on the maize foliar disease dataset. Overall, the proposed CKM-YOLO11 achieves a favorable balance between detection accuracy and model complexity. Specifically, CKM-YOLO11 attains a Box Precision of 0.796, a Recall of 0.747, an mAP@50 of 0.815, an mAP@50–95 of 0.484, and an F1-score of 0.770, indicating strong overall detection performance.

Compared with the baseline YOLO11, CKM-YOLO11 improves mAP@50 from 0.783 to 0.815, mAP@50–95 from 0.450 to 0.484, and F1-score from 0.740 to 0.770, corresponding to gains of 3.2, 3.4, and 3.0 percentage points, respectively. These results indicate that the proposed improvements effectively enhance the model’s ability to identify and localize disease targets.

In terms of model complexity, CKM-YOLO11 contains 2.72 M parameters and requires 6.5 GFLOPs, while still maintaining a lightweight design overall. Although its parameter count is slightly higher than that of YOLO11 (2.58 M), the computational cost increases only marginally. This suggests that the proposed method improves detection performance without introducing an excessive computational burden. Compared with larger models such as YOLOv3 and YOLOv6, CKM-YOLO11 shows clear advantages in terms of parameter count and computational cost while maintaining a high detection accuracy, demonstrating better overall efficiency.

**Figure 10:**
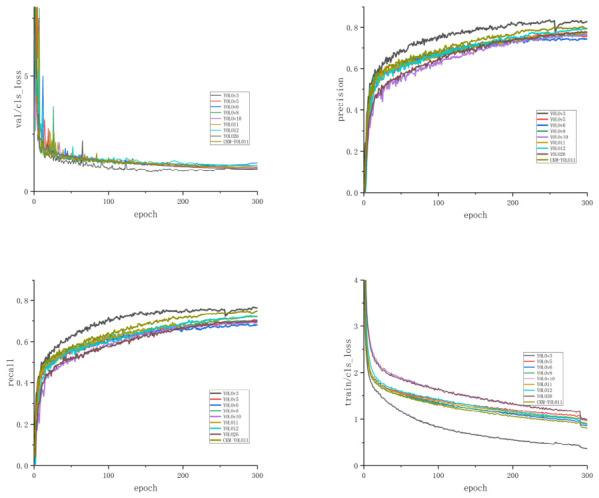
Curves of precision, recall, and classification loss during training for different models. The legends correspond to the evaluated model names shown in the curves.

**Figure 11:**
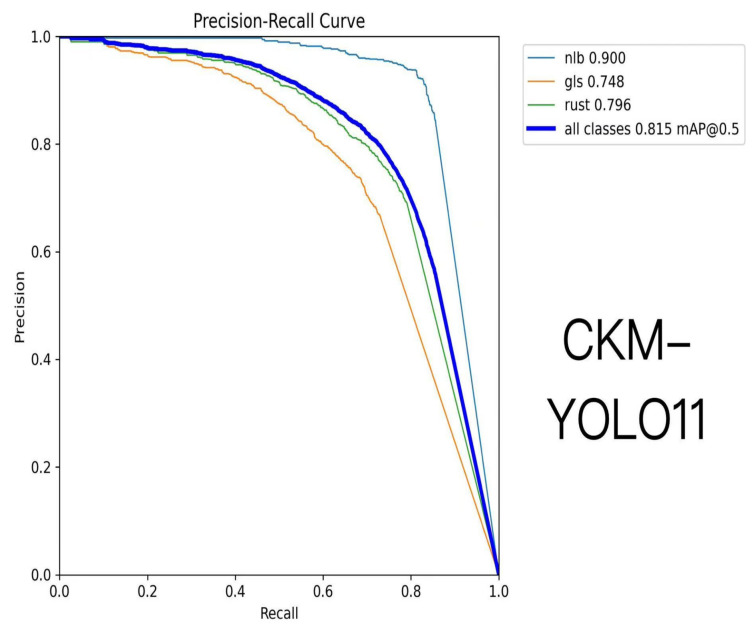
P-R curve of the improved model.

**Figure 12:**
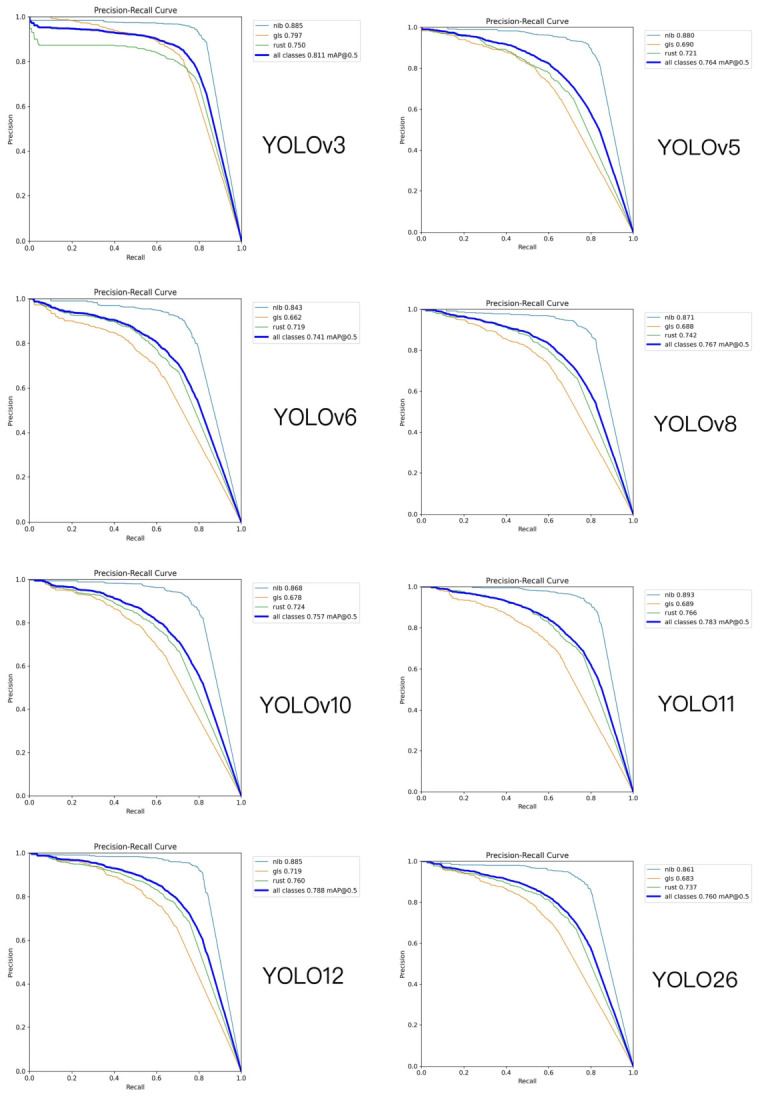
P-R curves of different baseline models.

The superior performance of CKM-YOLO11 under complex field backgrounds can be mainly attributed to the improved representation of local texture information and key lesion features. On the one hand, the C3k2-MLCA module embedded in the backbone enhances the ability of shallow features to capture small lesion regions. On the other hand, the lightweight residual attention structure at the P5 stage of the neck alleviates deep feature degradation, thereby improving the model’s discriminative ability for disease categories that are easily affected by background interference, such as gray leaf spot and leaf rust. These results demonstrate that the proposed method can effectively improve the accuracy and robustness of maize foliar disease detection while preserving the lightweight nature of the model. The training curves of different models are shown in Figure 10. The P-R curves of different models are shown in Figure 11 and Figure 12.

The comparison with mainstream YOLO-series models further shows that the proposed model does not merely pursue the maximum value of a single performance metric, but instead seeks a more practical balance among detection accuracy, model size, and deployment potential. For agricultural vision tasks, such a balance is of greater practical value than pursuing improvements in mAP alone, because the intended application scenarios include not only offline evaluation but also future deployment on agricultural monitoring devices, low-altitude inspection platforms, and field mobile terminals.

### 3.5. Visual Analysis

To further validate the effectiveness of the proposed model for maize foliar disease detection, a qualitative analysis was conducted from three perspectives: comparison of detection results, confusion matrix analysis, and Grad-CAM heatmap visualization. These visual analyses not only enable a qualitative comparison of different models on the three disease categories, namely rust, gls, and nlb, but also help reveal the advantages of the proposed model in terms of feature attention and discriminative mechanisms.

#### 3.5.1. Comparative Analysis of Detection Results of Different Models

**Figure 13:**
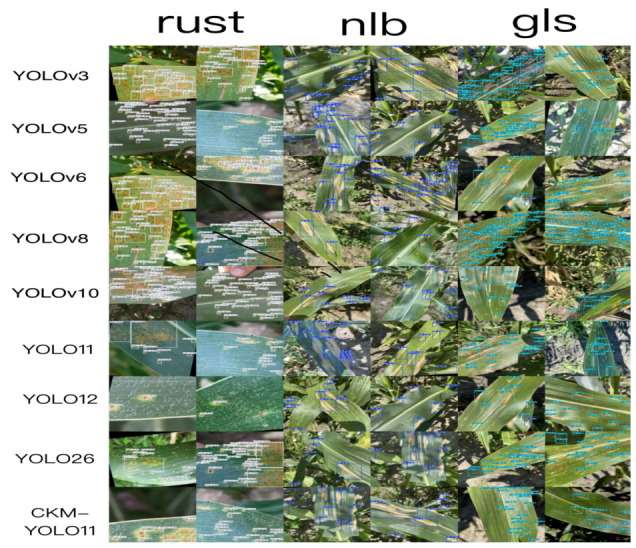
Comparison of detection results of different models on typical disease samples. Colored bounding boxes indicate detected disease regions, and labels show the predicted disease category and confidence score.

Figure 13 presents the detection results of different YOLO-series models and the proposed CKM-YOLO11 on representative maize leaf disease samples. The figure includes detection results for rust, gls, and nlb, with the predicted category and confidence score displayed next to each predicted bounding box. By comparing different disease samples horizontally and different models vertically, the differences in lesion localization, classification performance, and adaptability to complex backgrounds can be observed more intuitively.

Overall, as the YOLO series has evolved, the detection performance of these models on maize foliar disease images has gradually improved. Among the baseline models, YOLO11 already shows strong localization capability and detection accuracy. However, compared with the baseline models, the proposed CKM-YOLO11 yields more stable detection results for rust, gls, and nlb, especially in cases involving small lesions, blurred lesion boundaries, and complex leaf background textures.

For rust, lesions usually appear as numerous small and scattered spots, making them highly susceptible to interference from leaf textures and imaging noise. As shown in Figure 13, earlier models such as YOLOv3 and YOLOv5 are more prone to missed detections on such samples, and some small lesions are not effectively identified. Although YOLOv6, YOLOv8, YOLOv10, and YOLO11 exhibit improved detection performance, they may still produce local omissions or relatively low confidence scores in scenarios with dense small lesions. In contrast, the proposed model responds more completely to small rust lesions, covers diseased regions on the leaf surface more fully, and shows better detection stability.

For gls, lesions usually appear as strip-like, elongated fusiform, or grayish-brown patches. Because these lesions are partially similar to leaf veins and leaf textures, they are more likely to cause false detections or bounding-box offsets. Figure 13 shows that some baseline models produce incomplete localization, shifted bounding boxes, or misclassify leaf vein textures as lesions on gls samples. By contrast, the proposed model produces bounding boxes that are closer to the actual lesion regions and better distinguishes strip-like lesions from normal leaf vein textures, indicating stronger representation capability for medium-scale elongated disease features.

For nlb, lesions are relatively large and usually exhibit fusiform or irregularly expanded patterns, sometimes with blurred edges and gradual color transitions. As shown in Figure 13, YOLO-series models generally perform better on nlb than on rust. However, inaccurate localization or unstable confidence scores may still occur when lesion boundaries are unclear or when the background is complex. In contrast, the proposed model covers large-scale nlb lesions more completely and responds more reasonably to both the main lesion regions and their surrounding boundaries, thereby showing better localization accuracy.

Taken together, the results in Figure 13 indicate that the proposed model achieves better detection performance on most representative samples of rust, gls, and nlb. This demonstrates that the proposed improvements not only enhance recognition of small targets and fine-grained lesions, but also strengthen feature extraction and target localization under complex background conditions.

#### 3.5.2. Comparative Analysis of Confusion Matrices

**Figure 14:**
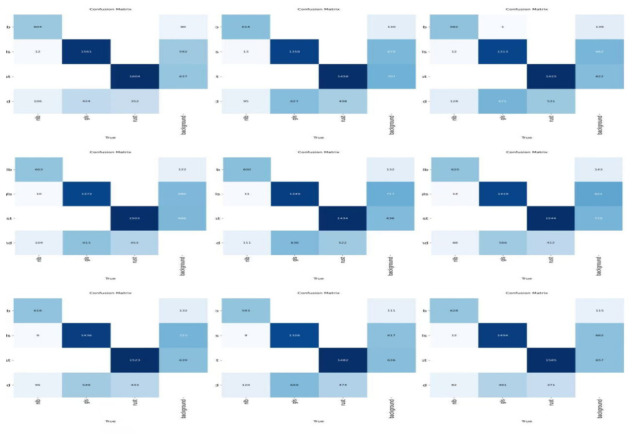
Comparison of confusion matrices of different models. Darker colors indicate larger sample counts, and diagonal cells represent correct predictions for each category.

Figure 14 presents the confusion matrices of YOLOv3, YOLOv5, YOLOv6, YOLOv8, YOLOv10, YOLO11, YOLO12, YOLO26, and the proposed CKM-YOLO11 on the test set. Compared with a simple visualization of detection results, confusion matrices provide a more intuitive view of the class-wise discrimination characteristics of different models. In particular, they are useful for analyzing the confusion among nlb, gls, rust, and background, thereby revealing the major error sources under complex field backgrounds.

Overall, the major errors of these models are not concentrated in direct confusion among disease categories. Instead, they mainly arise from confusion between disease targets and the background class. In other words, the core difficulty of this task does not primarily lie in distinguishing nlb, gls, and rust from one another, but in overcoming the strong interference caused by complex field backgrounds, vein textures, shadow regions, and weak lesion signals. This phenomenon is evident across all confusion matrices, where the off-diagonal errors associated with background are generally more prominent than the direct inter-class errors among disease categories. This indicates that the main bottleneck of maize foliar disease detection lies in insufficient background suppression and the tendency for weak lesion features to be suppressed during feature propagation, which is consistent with the design objectives of the proposed modules.

A further comparison between the baseline YOLO11 and the proposed CKM-YOLO11 shows that the improved model exhibits stronger concentration along the main diagonal for all three disease categories, indicating more stable classification results. Specifically, YOLO11 correctly identifies 620, 1419, and 1544 samples of nlb, gls, and rust, respectively, whereas CKM-YOLO11 increases these values to 628, 1494, and 1585. At the same time, the numbers of real disease samples misclassified as background by YOLO11 are 88, 566, and 412, respectively, which are reduced to 82, 491, and 371 by CKM-YOLO11. These results indicate that the improved model has a clearer advantage in reducing missed detections, especially for gls and rust, where the background-related errors decrease more noticeably. This suggests that the model’s ability to detect elongated gray leaf spot lesions and small rust lesions has been further enhanced.

From the perspective of background-induced false responses, CKM-YOLO11 still produces some cases in which background regions are predicted as disease categories. However, its overall error distribution is more balanced than that of YOLO11. Combined with the stronger main-diagonal concentration and reduced background-related errors, these results suggest that the improved model does not simply trade more false positives for higher sensitivity. Instead, it strengthens responses to real lesion regions while maintaining background suppression as much as possible. For the detection of small lesions and weak disease signals in natural field scenarios, this tendency to reduce the risk that real lesions are suppressed and misclassified as background is of substantial practical significance.

From the perspective of different disease categories, nlb shows relatively limited direct inter-class confusion across models, but its detection results are still affected by complex background regions. gls, because of its elongated shape and similarity to leaf veins, shows relatively high confusion with the background in several models. rust, due to its small target size and scattered distribution, imposes higher demands on local detail modeling. Therefore, the improvement achieved by the proposed model on gls and rust more strongly demonstrates that the introduced attention mechanisms indeed enhance the network’s ability to distinguish local lesion textures from complex backgrounds.

These improvements can be attributed to two main factors. First, the C3k2-MLCA module in the backbone strengthens the joint modeling of local texture details and global contextual information during shallow feature extraction, enabling the network to distinguish real lesions from background noise at an earlier stage. Second, the MLCA-HeadLite residual module at the P5 stage alleviates excessive suppression of weak lesion responses in deep features through a gentler reweighting mechanism, thereby reducing the risk that small lesions are misclassified as background during deep feature propagation. Overall, the enhanced main-diagonal responses and reduced background confusion observed in the confusion matrices confirm that the proposed method provides stronger disease discrimination and better robustness under complex background conditions.

#### 3.5.3. Grad-CAM Heatmap Visualization Analysis

**Figure 15:**
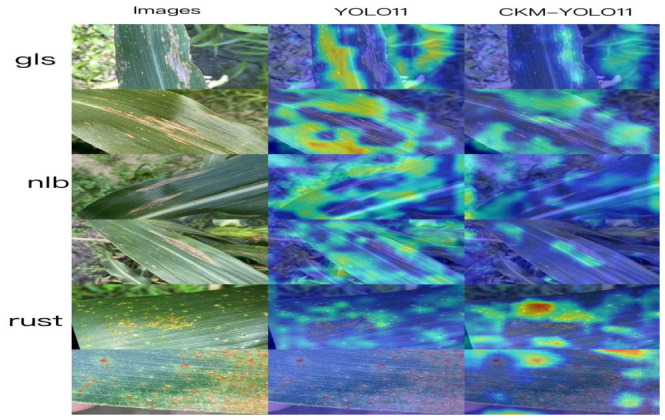
Comparison of Grad-CAM heatmaps between YOLO11 and CKM-YOLO11. Warmer colors indicate stronger activation responses, and cooler colors indicate weaker responses.

To further reveal the differences in feature attention between models for maize foliar disease recognition, typical samples of gray leaf spot (gls), northern leaf blight (nlb), and leaf rust (rust) were selected for Grad-CAM heatmap visualization analysis of the baseline YOLO11 and the proposed CKM-YOLO11, as shown in Figure 15. As a representative gradient-based class activation mapping method, Grad-CAM provides interpretable support for identifying the discriminative regions used by convolutional neural networks for category prediction [25]. In the figure, the first column shows the original input images, the second column shows the heatmaps generated by YOLO11, and the third column shows the heatmaps generated by CKM-YOLO11. By comparing the response distributions of different models over lesion regions, leaf-vein textures, and background areas, the advantages of the improved model in feature extraction and target attention can be more intuitively analyzed.

From the overall visualization results, the high-response regions of the baseline YOLO11 are relatively scattered. In addition to real lesions, obvious responses are also observed in leaf-vein textures, leaf edges, and some background regions, indicating a certain degree of attention diffusion under complex field backgrounds. By contrast, the heatmap responses of CKM-YOLO11 are mainly concentrated on the lesion body, and the high responses in non-diseased regions are markedly reduced. In the last rust sample, the Grad-CAM heatmap of baseline YOLO11 fails to clearly highlight the lesion area, whereas CKM-YOLO11 shows obvious activation at the true lesion positions, indicating stronger attention to weak, small, and scattered lesions. These results suggest that CKM-YOLO11 can effectively suppress background interference and focus the discriminative responses on the true diseased regions, which is consistent with the design objective of enhancing local texture modeling while reducing background noise interference.

For gls samples, lesions usually appear as elongated strip-like structures extending along the leaf veins and are therefore highly similar to normal vein textures, making feature confusion likely. The results show that YOLO11 produces strong activations not only over lesion regions but also in surrounding background areas and along extended vein regions, indicating insufficient modeling of the differences between elongated lesions and normal leaf textures. In contrast, CKM-YOLO11 concentrates its responses more precisely on the lesion stripes themselves, with substantially reduced irrelevant activations in the background, suggesting that the improved model can better distinguish gray leaf spot lesions from normal vein textures.

For nlb samples, lesions are usually larger and often exhibit fusiform or irregular expansion patterns, with blurred boundaries and gradual color transitions. As shown in the figure, although YOLO11 can roughly attend to the lesion region, its high-response areas are more diffuse and tend to spread into the surrounding healthy leaf surface, indicating insufficient boundary localization. By contrast, CKM-YOLO11 shows stronger regional focus on nlb samples, with responses concentrated more on the lesion body and more continuous activations around the lesion contours, suggesting better boundary perception and regional discrimination for medium- to large-scale lesions.

For rust samples, lesions usually appear as numerous small and scattered spots, which place higher demands on the model’s ability to extract local details. The heatmaps show that YOLO11 produces relatively dispersed responses on rust samples, and some high-response areas do not accurately fall on the rust spots but are instead influenced by nearby leaf textures or background regions. In contrast, CKM-YOLO11 produces more prominent responses to small rust lesions and forms clearer activation centers over multiple fine rust spots, indicating stronger sensitivity and stability in the recognition of tiny lesion targets. This further verifies the effectiveness of the introduced modules in enhancing shallow local-detail features.

Taken together, the Grad-CAM visualizations of the three disease categories show that, compared with baseline YOLO11, the proposed CKM-YOLO11 can focus attention more accurately on true lesion regions while effectively reducing erroneous responses to background textures, leaf-vein structures, and irrelevant areas. This can be mainly attributed to two factors. On the one hand, the C3k2-MLCA module in the backbone strengthens joint modeling of local texture details and global semantic information during shallow feature extraction, enabling the model to distinguish subtle differences between lesions and background at an earlier stage. On the other hand, the MLCA-HeadLite module in the P5 neck alleviates the excessive suppression of weak lesion responses in deep features through residual reweighting, thereby allowing the model to maintain effective attention to weak lesions even under complex background conditions. These results indicate that the improved model not only outperforms the baseline in detection accuracy, but also exhibits stronger specificity and interpretability in its feature-attention mechanism.

In response to background interference, the comparison between YOLO11 and CKM-YOLO11 further indicates that the proposed model can reduce erroneous attention to non-lesion regions such as dry leaf edges, soil-colored background areas, shadows, and normal vein textures. In the baseline YOLO11 heatmaps, high-response regions are more likely to spread beyond the lesion body and partially cover surrounding background textures. By contrast, CKM-YOLO11 produces more concentrated activations around true lesion regions, especially for small rust spots and elongated gray leaf spot lesions. This suggests that the introduced C3k2-MLCA module improves local lesion texture perception in the backbone, while the MLCA-HeadLite residual attention design helps preserve weak lesion responses without amplifying irrelevant background noise. Therefore, the visual comparison provides qualitative evidence that the proposed modules contribute to both background suppression and weak-lesion retention under complex field conditions.

The visualization results further provide qualitative support for the effectiveness of the proposed method. Compared with the baseline model, CKM-YOLO11 exhibits more concentrated lesion-response regions and fewer erroneous background responses in detection results, confusion matrices, and Grad-CAM heatmaps, indicating that the improved modules indeed enhance the model’s attention to true lesion regions and, to some extent, validate the intended design goals of local texture enhancement and background suppression.

### 3.6. Extending Detection Results to Field Management Support

To further demonstrate the practical value of the model in real field scenarios, this study extends the maize foliar disease detection results to a disease severity representation framework and explores their potential conversion into field management information. It should be noted that the severity grades and corresponding management suggestions presented in this section are auxiliary application-oriented interpretations based on the detection results. Their purpose is to illustrate the feasibility of extending model outputs toward field management support, rather than to provide standardized pathological diagnoses or fixed pesticide prescriptions. Compared with conventional outputs that only provide disease categories and bounding-box locations, this extension allows the model output to move beyond the question of whether a disease is detected toward how the disease severity can be expressed, thereby providing useful references for field inspection, risk identification, and subsequent management prioritization.

#### 3.6.1. Purpose of Constructing Disease Severity Maps

The purpose of introducing disease severity representation is to improve the interpretability, hierarchy, and field-level readability of the model outputs. First, disease categories and bounding-box locations alone can indicate the presence of a target, but cannot directly reflect the severity level of the current sample, which is often an important factor in determining management priority. Second, under natural field conditions, different diseases vary substantially in morphology, scale, and spatial distribution. Organizing the detection results into severity levels such as mild, moderate, and severe therefore helps improve the intuitiveness and interpretability of model outputs. Third, from the perspective of practical applications, mapping detection results to management levels such as continuous monitoring, priority re-inspection, and priority intervention makes the outputs more relevant to field scouting and agricultural management scenarios.

**Figure 16:**
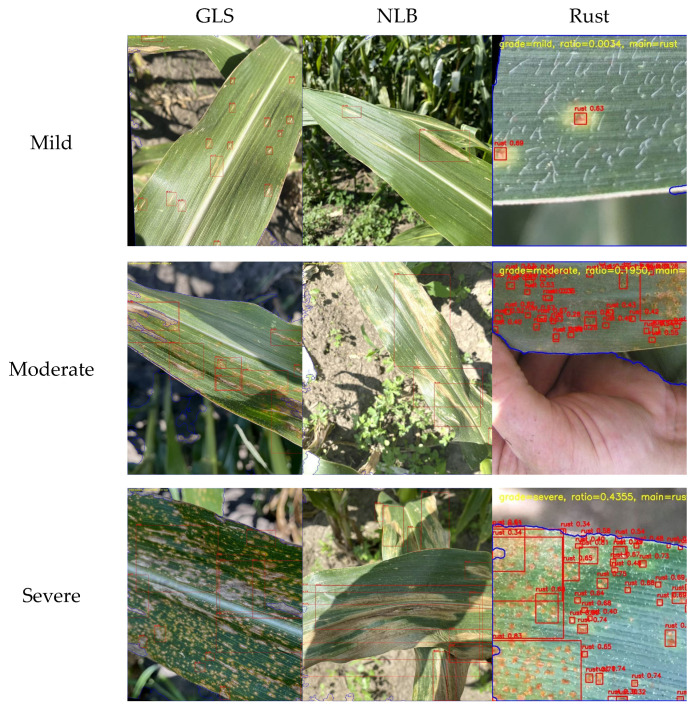
Schematic diagram of disease severity grading based on detection results. Rows indicate severity levels, columns indicate disease categories, and boxes show representative examples used for severity representation.

Based on these considerations, severity grading was not designed as an independent learning task, nor was an additional disease severity classification network constructed. Instead, a lightweight and interpretable severity representation process was developed based on the detection results of the improved model CKM-YOLO11 by quantifying the area relationship between lesion regions and the main visible leaf region. The purpose of this design is not to establish a strict pathological grading system, but rather to demonstrate the application potential of extending object detection outputs to management-oriented information. The schematic examples of the three severity levels are shown in Figure 16.

#### 3.6.2. Grading Rules and Implementation Procedure

In this study, the ratio of lesion area to main leaf area was adopted as the quantitative indicator of disease severity, denoted as ratio. Based on this ratio, disease severity was divided into three levels: mild (0 < ratio ≤ 10%), moderate (10% < ratio ≤ 35%), and severe (ratio > 35%). Here, ratio represents the proportion of the effective lesion area within the visible main leaf area. It should be emphasized that these thresholds were not directly copied from any single official pathological standard. Instead, they were established as an engineering-oriented grading rule with reference to the commonly used idea that foliar disease severity is often expressed as the percentage of damaged leaf area, and were further adapted to the image post-processing pipeline used in this study [2,11]. Therefore, their essential purpose is to provide a structured representation of detection results rather than to replace expert scoring systems.

The implementation procedure is as follows. First, the trained CKM-YOLO11 model is used to detect disease targets in the input image and obtain disease bounding boxes for all categories. Second, the visible main leaf region is extracted from the entire image to serve as the denominator for severity calculation. Specifically, a main-leaf mask is constructed by combining HSV-based wide-threshold segmentation, Excess Green (ExG) vegetation enhancement, morphological processing, and connected-component screening. Third, all disease detection boxes produced by the model are merged into a union mask to avoid repeated accumulation of lesion areas when multiple detection boxes overlap. Next, the union mask is intersected with the main leaf region to obtain the effective lesion region, from which the lesion-to-leaf area ratio is computed. Finally, the sample is assigned to one of the three severity levels, namely mild, moderate, or severe, according to the ratio threshold defined above.

Compared with simple box-by-box area accumulation, this method uses the whole visible main leaf as the denominator and the union lesion region as the numerator, which provides a more reasonable image-level representation of disease severity. At the same time, because the entire process is based directly on detection outputs, it has the advantages of simple implementation, clear interpretability, and natural compatibility with the object detection model. This makes it suitable as a post-processing strategy for converting detection results into application-oriented information. It should also be noted that this study does not include a comparative experiment on severity grading between the original model and the improved model. Therefore, the main focus of this section is to demonstrate the extensible application of CKM-YOLO11 outputs, rather than to compare the merits of different severity grading systems themselves.

#### 3.6.3. Management Priority and Auxiliary Control Recommendations

Based on the above severity representation, the three severity levels of mild, moderate, and severe were further mapped to different management priorities to illustrate the potential application of detection results in field-assisted decision-making. Specifically, mild samples may be regarded as samples requiring continuous monitoring, meaning that disease has been detected but the currently affected area is still relatively limited, making such samples more suitable for subsequent routine inspection or shortened re-inspection intervals. Moderate samples may be regarded as samples requiring priority re-inspection, indicating that the disease has already exerted a more noticeable influence on the functional leaf region and should therefore receive more attention in field management, with further judgment made in combination with disease expansion trends, meteorological conditions, and crop growth stages. Severe samples may be regarded as samples requiring priority intervention, indicating that the image already shows a relatively high level of lesion coverage and should therefore be prioritized for on-site verification and management response.

It should be emphasized that the above interpretation of continuous monitoring–priority re-inspection–priority intervention is intended to provide a more application-oriented explanatory framework for model outputs, rather than to directly generate standardized pesticide prescriptions. For maize foliar diseases, actual control decisions still need to be made based on a comprehensive assessment of the disease type, crop growth stage, regional weather conditions, disease development speed, and local plant protection recommendations. Therefore, this study does not provide fixed pesticide combinations, unified dosages, or standardized operational procedures. Instead, the emphasis is placed on illustrating how detection results can support management prioritization at the application level.

From an application perspective, this severity representation can improve the readability of object detection outputs by extending them beyond simple category–location information to include additional severity–priority information. For use cases such as UAV-based field scouting, mobile disease inspection, and intelligent field monitoring devices, this extension is practically meaningful because it reduces the abstraction of raw detection outputs and provides a more intuitive basis for manual re-inspection, risk screening, and regional disease-status statistics [2,16,19].

Based on this severity representation, this study further provides auxiliary control recommendations oriented toward field applications. For mild samples, the lesion coverage is relatively low, and the result is more suitable for guiding continued monitoring or shortening the interval between re-inspections; immediate chemical intervention is generally not recommended solely on the basis of a single image result. For moderate samples, the disease has already caused a relatively obvious impact on the leaf functional region, and whether further control measures should be initiated may be evaluated in combination with persistent humid weather, disease expansion trends, and critical maize growth stages. For severe samples, the image indicates a relatively high level of damage and such samples should be prioritized for on-site verification and management response. It should again be emphasized that these recommendations are intended only to demonstrate the feasibility of extending detection outputs into field management information and do not constitute fixed pesticide prescriptions. The specific pesticide type, dosage, application timing, and application frequency should still be determined according to local plant protection recommendations, regional disease-warning information, and registered label instructions.

#### 3.6.4. Scope and Limitations of This Extension

Although the above severity representation is feasible from both engineering and application perspectives, its scope of use should be clearly defined. First, the proposed grading results are derived from bounding-box-based post-processing and leaf-region estimation, rather than from pixel-level lesion segmentation or expert-annotated pathological grading standards. Therefore, the resulting severity levels are more appropriate as an engineering-oriented severity representation than as strict pathological diagnostic conclusions. Second, the current process is based only on the outputs of the improved model CKM-YOLO11 and has not yet been systematically compared with the original model or with expert disease scoring. Accordingly, this section should be understood as an application extension, rather than as a core methodological validation. Finally, the mapping from detection results to management suggestions remains an auxiliary decision-support process whose purpose is to improve the practical readability of model outputs rather than to replace expert judgment in field management.

Overall, by introducing disease severity representation on top of detection results, this study extends the output of CKM-YOLO11 from a conventional object detection result to a more application-oriented form of information that can support field scouting and management prioritization. This attempt suggests that disease detection models for complex environments may serve not only for disease identification, but also for disease interpretation and management support.

## 4. Discussion

### 4.1. Interpretation of Results

The results of this study indicate that, under complex natural field backgrounds, the main challenge of maize foliar disease detection does not lie solely in the direct discrimination among disease categories. Rather, it is more strongly associated with the visual confusion between lesion regions and background textures, as well as the tendency of weak lesion responses to be suppressed during deep feature propagation. The proposed CKM-YOLO11 improves the model’s representation capability for disease targets in complex backgrounds from two aspects: by introducing the C3k2-MLCA module into the backbone, it strengthens shallow local texture modeling; and by introducing the MLCA-HeadLite module at the P5 stage, it enhances the preservation of weak lesion features in deep layers. The results of the ablation experiments, deployment-position comparison experiments, and visualization analyses consistently demonstrate that the improvements reported in this study are not incidental gains resulting from the simple accumulation of individual modules, but rather arise from the coordinated optimization of different structural stages of the network.

### 4.2. Limitations

At the same time, the proposed method still has several limitations. First, the current dataset mainly consists of leaf images collected from a specific region and under limited imaging conditions, and the generalization ability of the model across different regions, years, and acquisition devices still requires further verification. Second, the disease severity grading proposed in this study is an engineering-oriented post-processing representation built upon the detection results. Since no external references such as expert scoring or pixel-level lesion segmentation were introduced, it is more appropriate to regard this component as an application extension rather than a strict pathological diagnostic conclusion. In future work, more systematic studies could be conducted on the field deployment adaptability of the model by incorporating low-altitude UAV close-range imaging, mobile-device acquisition, and multimodal sensing approaches.

In addition, although the proposed model was evaluated under complex field backgrounds containing variations in illumination, leaf posture, shadows, and background interference, more extreme field conditions remain challenging. For example, severe leaf overlap may obscure lesion boundaries and reduce the visibility of small disease spots, while motion blur caused by UAV-based or fast mobile imaging may weaken texture details that are important for disease discrimination. Under such conditions, the model may still suffer from missed detections or less precise localization. Future work will therefore further evaluate CKM-YOLO11 using UAV-acquired and motion-blurred field images, and may incorporate image deblurring, temporal information, or multi-view data acquisition to improve robustness under more challenging agricultural monitoring scenarios.

## 5. Conclusions

To address the two major challenges in maize foliar disease detection under complex natural field backgrounds, namely background confusion and loss of weak lesion responses, this study proposes an improved lightweight detection model termed CKM-YOLO11, which is based on YOLO11. By introducing the C3k2-MLCA module into the backbone, the model strengthens the joint modeling of local texture details and global contextual information in shallow layers. In addition, by introducing the MLCA-HeadLite residual module at the P5 stage, the model alleviates the tendency of weak lesion responses to be excessively suppressed during deep feature fusion.

Experimental results demonstrate that CKM-YOLO11 achieves strong overall performance on the self-constructed maize disease dataset with complex field backgrounds. Compared with the baseline YOLO11, the proposed model improves both mAP@50 and mAP@50–95, while also showing stronger disease discrimination capability and better detection robustness under complex background conditions. Furthermore, the effectiveness of the proposed model in background suppression and weak lesion preservation is supported by the confusion matrix analysis, Grad-CAM visualizations, and detection result comparisons. In addition, this study further extends the detection outputs to disease severity representation and management-priority information, demonstrating the potential of object detection models for application-oriented field support. Overall, this work provides a valuable reference for the lightweight design and application extension of maize disease detection models in complex field environments, and is also consistent with recent development trends in field-scene analysis, edge deployment, and interpretable agricultural vision systems [2,16,19,25].

## Figures and Tables

**Table 1:** Hyperparameter settings for model training.

Parameter	Value
Image size	640
Batch size	8
Epochs	300
Learning rate	0.01
Momentum	0.937
Weight decay	0.0005
Optimizer	SGD

**Table 2:** Experimental environment configuration.

Hardware/Software	Model/Version
GPU	NVIDIA GeForce RTX 4060 Laptop GPU (8 GB; NVIDIA Corporation, Santa Clara, CA, USA)
Operating System	Windows 11 (Microsoft Corporation, Redmond, WA, USA)
CUDA	12.1 (NVIDIA Corporation, Santa Clara, CA, USA)
Python	3.12
PyTorch	2.5.1
Visual Studio Code	1.113.0 (Microsoft Corporation, Redmond, WA, USA)

**Table 3:** Results of the comparison experiment on different head deployment positions.

Model	Box	R	mAP@50	mAP@50–95	F1-Score	GFLOPs	Params/M	FPS
YOLO11	0.763	0.719	0.783	0.450	0.740	6.3	2.58	322.58
P3	0.798	0.739	0.808	0.480	0.770	6.4	2.59	285.71
P4	0.799	0.726	0.802	0.473	0.760	6.4	2.62	263.16
P5	0.804	0.725	0.807	0.476	0.760	6.4	2.59	344.83
P3 + P4	0.780	0.736	0.799	0.472	0.760	6.6	2.63	263.16
P3 + P5	0.789	0.735	0.801	0.473	0.760	6.4	2.59	333.33
P4 + P5	0.809	0.720	0.803	0.471	0.760	6.5	2.62	344.83
P3 + P4 + P5	0.789	0.741	0.804	0.475	0.760	6.6	2.63	135.14

**Table 4:** Results of the ablation study.

Model	Box	R	mAP@50	mAP@50–95	F1-Score	Param/M	FPS
YOLO11	0.763	0.719	0.783	0.450	0.740	2.58	322.58
YOLO11 + C3k2-MLCA	0.808	0.744	0.811	0.478	0.770	2.72	344.83
YOLO11 + MLCA-HeadLite (P3)	0.798	0.739	0.808	0.480	0.770	2.59	285.71
YOLO11 + MLCA-HeadLite (P3) + C3k2-MLCA	0.785	0.738	0.804	0.477	0.770	2.73	263.16
YOLO11 + MLCA-HeadLite (P3 + P5)	0.789	0.735	0.801	0.473	0.760	2.59	333.33
YOLO11 + MLCA-HeadLite (P3 + P5) + C3k2-MLCA	0.800	0.738	0.806	0.472	0.760	2.73	263.16
YOLO11 + MLCA-HeadLite (P5)	0.804	0.725	0.807	0.476	0.760	2.59	344.83
CKM-YOLO11	0.796	0.747	0.815	0.484	0.770	2.72	294.12

**Table 5:** Comparison of detection performance of different YOLO-series models on the maize foliar disease dataset.

Model	Box	R	mAP@50	mAP@50–95	F1-Score	Weight/MB	GFLOPs	Param/M
YOLOv3	0.829	0.756	0.811	0.549	0.790	20.6	282.2	103.7
YOLOv5	0.756	0.702	0.764	0.432	0.730	5.3	7.1	2.51
YOLOv6	0.703	0.713	0.741	0.418	0.710	17.5	11.7	4.23
YOLOv8	0.77	0.696	0.767	0.435	0.730	12.6	8.1	3.01
YOLOv10	0.751	0.688	0.757	0.429	0.720	5.8	6.5	2.27
YOLO11	0.763	0.719	0.783	0.450	0.740	5.5	6.3	2.58
YOLO12	0.793	0.715	0.788	0.456	0.750	11.2	6.3	2.56
YOLO26	0.767	0.694	0.76	0.431	0.730	5.5	5.2	2.38
CKM-YOLO11	0.796	0.747	0.815	0.484	0.770	11.5	6.5	2.72

## Data Availability

Data are available upon request due to privacy or ethical restrictions. Data from this study are available from the corresponding authors upon request. Because of the privacy implications of the data in this study, these data are not publicly available.

## References

[1] Shafay M., Hassan T., Owais M., Hussain I., Khawaja S.G., Seneviratne L., Werghi N. (2025). Recent advances in plant disease detection: Challenges and opportunities. Plant Methods.

[2] Pfordt A., Paulus S. (2025). A review on detection and differentiation of maize diseases and pests by imaging sensors. J. Plant Dis. Prot..

[3] Timilsina S., Sharma S., Kondo S. (2025). Advancements in maize leaf disease detection, segmentation and classification: A review. Biosyst. Eng..

[4] Zhong T., Zhu M., Zhang Q., Zhang Y., Deng S., Guo C., Xu L., Liu T., Li Y., Bi Y. (2024). The ZmWAKL-ZmWIK-ZmBLK1-ZmRBOH4 module provides quantitative resistance to gray leaf spot in maize. Nat. Genet..

[5] Ijaz B., Fan X. (2024). Understanding Northern Corn Leaf Blight (NCLB) Disease Resistance in Maize: Past Developments and Future Directions. Plant Stress.

[6] Qian X., Zhang C., Chen L., Li K. (2022). Deep learning-based identification of maize leaf diseases is improved by an attention mechanism: Self-attention. Front. Plant Sci..

[7] Pacal I., Kunduracioglu I., Alma M.H., Deveci M., Kadry S., Nedoma J., Slany V., Martinek R. (2024). A systematic review of deep learning techniques for plant diseases. Artif. Intell. Rev..

[8] Hughes D.P., Salathe M. (2015). An open access repository of images on plant health to enable the development of mobile disease diagnostics. arXiv.

[9] Mohanty S.P., Hughes D.P., Salathe M. (2016). Using deep learning for image-based plant disease detection. arXiv.

[10] Singh D., Jain N., Jain P., Kayal P., Kumawat S., Batra N. (2019). PlantDoc: A dataset for visual plant disease detection. arXiv.

[11] Ahmad A., Saraswat D., El Gamal A., Johal G. (2021). CD&S dataset: Handheld imagery dataset acquired under field conditions for corn disease identification and severity estimation. arXiv.

[12] Shui Y., Yuan K., Wu M., Zhao Z. (2024). Improved Multi-Size, Multi-Target and 3D Position Detection Network for Flowering Chinese Cabbage Based on YOLOv8. Plants.

[13] Fu Y., Zhang Y. (2025). Lightweight Rice Leaf Disease Detection Method Based on Improved YOLOv8. Eng. Lett..

[14] Gülmez B. (2024). Advancements in maize disease detection: A comprehensive review of convolutional neural networks. Comput. Biol. Med..

[15] Li R., Li Y., Qin W., Abbas A., Li S., Ji R., Wu Y., He Y., Yang J. (2024). Lightweight network for corn leaf disease identification based on improved YOLO v8s. Agriculture.

[16] Nakatumba-Nabende J., Murindanyi S. (2025). Deep learning models for enhanced in-field maize leaf disease diagnosis. Mach. Learn. Appl..

[17] Ultralytics YOLO11 Documentation. https://docs.ultralytics.com/zh/models/yolo11/.

[18] Apicella A., Isgrò F., Prevete R. (2025). Don’t push the button! Exploring data leakage risks in machine learning and transfer learning. Artif. Intell. Rev..

[19] Upadhyay A., Chandel N.S., Singh K.P., Chakraborty S.K., Nandede B.M., Kumar M., Subeesh A., Upendar K., Salem A., Elbeltagi A. (2025). Deep learning and computer vision in plant disease detection: A comprehensive review of techniques, models, and trends in precision agriculture. Artif. Intell. Rev..

[20] Wan D., Lu R., Shen S., Xu T., Lang X., Ren Z. (2023). Mixed local channel attention for object detection. Eng. Appl. Artif. Intell..

[21] Hu J., Shen L., Sun G. Squeeze-and-Excitation Networks. Proceedings of the IEEE/CVF Conference on Computer Vision and Pattern Recognition.

[22] Woo S., Park J., Lee J.Y., Kweon I.S. CBAM: Convolutional Block Attention Module. Proceedings of the European Conference on Computer Vision.

[23] Wang Q., Wu B., Zhu P., Li P., Zuo W., Hu Q. ECA-Net: Efficient channel attention for deep convolutional neural networks. Proceedings of the IEEE/CVF Conference on Computer Vision and Pattern Recognition.

[24] Albahli S., Masood M. (2022). Efficient attention-based CNN network (EANet) for multi-class maize crop disease classification. Front. Plant Sci..

[25] Selvaraju R.R., Cogswell M., Das A., Vedantam R., Parikh D., Batra D. Grad-CAM: Visual explanations from deep networks via gradient-based localization. Proceedings of the IEEE International Conference on Computer Vision.

